# An investigation of methods to improve recall for the patient-reported outcome measurement in COPD patients: a pilot randomised control trial and feasibility study protocol

**DOI:** 10.1186/s40814-019-0475-9

**Published:** 2019-07-18

**Authors:** Sheree M. S. Smith, Stephen Jan, Joseph Descallar, Guy B. Marks

**Affiliations:** 1 School of Nursing and Midwifery, Western Sydney University, Penrith, Sydney, 2751 Australia; 2 George Institute for Global Health, University of New South Wales, Randwick, Sydney, Australia; 3 Ingham Applied Medical Research Institute, Liverpool, Sydney, Australia; 4 Woolcock Institute of Medical Research, Glebe, Sydney, Australia; 5 South Western Sydney Clinical School, University of New South Wales, Sydney, Australia

**Keywords:** Patient-reported outcomes, Recall bias, Patient-reported outcome measurement

## Abstract

**Background:**

Patient-reported outcomes (PRO) are used to measure the effectiveness of interventions for management of chronic conditions such as chronic obstructive pulmonary disease. Many of these instruments require respondents to describe the change in their health status from baseline to a follow-up assessment and poor recall of previous health status often limits the usefulness and validity of these PRO measures. The use of technology has recently increased in PRO measurement. This study aims to mitigate the problems of poor recall by evaluating different strategies as a way to improve the validity of recall of health status among adults with COPD.

**Methods:**

A pilot randomised controlled trial of three strategies to improve patient recall will be tested in an acute care clinical environment. The first strategy is the use of tablet computer technology’s audio-visual facility, the second strategy is the provision of base line PRO responses prior to patients completing their follow-up questionnaires and third is standard practice of completing a questionnaire independently of previous responses. The feasibility of conducting this study in a busy clinical environment will be ascertained using the NIHR criteria for assessing feasibility.

**Discussion:**

There is variability in a person’s ability to recall past events. With studies utilising patient-reported outcome measurement, it has become critically important to develop strategies and ways of supporting the patient to be more accurate recalling their health status. The adaptation of various technological features within mobile devices may provide an opportunity in clinical research studies to improve patient recall of their health status.

**Trial registration:**

ANZCTR12618001605280.

**Electronic supplementary material:**

The online version of this article (10.1186/s40814-019-0475-9) contains supplementary material, which is available to authorized users.

## Introduction

### Background and rationale

Chronic obstructive pulmonary disease (COPD) is a chronic illness that is known to cause significant disability and impair quality of life [1]. Patient-reported outcome (PRO) instruments, such as health-related quality of life questionnaires, are often used in COPD clinical trials [2]. The performance of these instruments is often described as having validity, reliability and responsiveness. Validity is deemed when an instrument or questionnaire contains a set of questions that accurately measures the phenomena such as quality of life. This validity is usually established through three stages; content validity, criterion validity and construct validity [3]. Whereas, reliability, often termed as reproducibility, occurs when an instrument repeatedly provides the same results in different patients with the same stable disease [4]. The responsiveness of a questionnaire is based on its ability to detect change over time, even if these changes are small [3]. The responsiveness of a questionnaire may be inadvertently affected when patients are required to respond to a questionnaire by self-report at different time points and rely on their memory of the experience or symptoms that have occurred [5].

The use of PRO instruments in clinical trials often requires the participant to respond (self-report) to questionnaires about subjective components of their health status at baseline and again at follow-up assessments. Recall of previous information and/or an experience is an important element in the successful utilisation of these PRO instruments in clinical trials [6]. However, patients’ recall is often imperfect. Recall problems fall into two types: random errors in recall and recall bias (p197) [7]. It has been acknowledged that people have varying ability to recall information. People with COPD are often over 50 years of age and the ability to accurately recall information appears to decline with age [8, 9]. In addition, people who have both anxiety and depression have been found to have impaired ability to accurately recall information [10]. Anxiety and depression more commonly occurs in people with COPD [1] and these comorbid conditions may affect the accuracy in recalling information or events [11] .

Recall bias is potentially important as it can affect the validity of a study [6, 11]. Recall bias occurs when participants in the study’s intervention arm may be questioned several times over a period of time about specific aspects of their condition [12]. For instance, a study that is evaluating a technique to improve the symptoms of COPD, patients may be asked about their breathlessness as part of an 8-week pulmonary rehabilitation intervention where they can observe themselves being able to complete physical activities more easily over time. In contrast, the participants in the control arm of the study are asked questions about their breathlessness at the beginning and end of the study. The control group receive usual care in between these data collection time points and the ability to recall any improvement may be reduced without a prompt to do so [12].

In addition to biased recall, errors in recall may also occur. People who complete questionnaires about their physical activity have been found to misreport and/or misinterpret questions [13]. Self-reported data acquired from questionnaires completed by people with impaired physical ability have been shown to be less reliable than those from fully fit people [11]. In the Adventist Health Study, participants’ recalling (self-reported) body weight underestimated the actual body weight [13]. Strategies to limit the impact of impaired recall have included the comparison of completed patient questionnaires with objective measurements [14] such as lung function, six-minute walk test and physical activity completed by patients and verified by observers.

To reduce poor recall, a potential strategy could be, at a subsequent data collection interval, to provide participants with access to their previously completed questionnaire responses. This strategy seeks to anchor [5] participants to their baseline responses during the completion of the questionnaire at a follow-up appointment to aid recall. More recently, various forms of technology have been used in the completion of patient-reported outcome instruments. Telemonitoring [15], web-based platforms [16], touch screens linked to electronic health records [17] and mobile applications [15] all form technology-based mechanisms for patients to engage in self-reporting aspects of their health through the inputting of their responses to specific questions. Therefore, a second strategy could be developed using tablet technology to improve patient recall. To our knowledge, the use of tablet technology’s audio-visual facility has not been utilised to enhance patient recall in the self-reporting of health status through visual and sound recognition from baseline to follow-up time intervals.

### Objectives and hypothesis

Our proposed research seeks to mitigate the problems of poor recall by piloting three different strategies concurrently and to assess the feasibility of these strategies in a busy hospital clinical environment. The first strategy is to evaluate the use of tablet computer technology’s audio-visual functionality as a way to improve the validity of recall of health status among trial participants with COPD. The method involves making an audio-visual recording of the participant’s initial responses to a quality of life questionnaire. Participants will be able to see their image and reflect on how they look at the baseline, and listen to how they sound prior to completing the follow-up questionnaires. This audio-visual recording will act as frame of reference and as an anchor to their past health status to aid their memory. The second strategy is the provision of access to the patient’s previous completed baseline questionnaire prior to completing their follow-up questionnaire. The third strategy is current practice of completing quality of life questionnaires independent of previous responses.

Specifically, we are seeking to understand among patients with COPD undergoing pulmonary rehabilitation, what is the change that occurs in patient-reported measurement when tablet computer technology is used to audio-visually record patients’ responses to health questionnaires on their subsequent ability to recall their previous health status? Secondly, we are assessing whether the provision of previous responses to quality of life questionnaires differs between COPD patients who are enrolled in pulmonary rehabilitation and those who are waiting to commence the program. Thirdly, we will compare the current practice of completing quality of life questionnaires before and after the program. Finally, we will be assessing the feasibility of conducting this study in a busy acute care clinical environment. Our hypothesis is that the magnitude of the difference (responsiveness) between groups of COPD patients will be greatest in those who saw the audio-visual recording.

## Methods

This is a pilot randomised control parallel group trial of three strategies to improve patient recall in the self-report of health status using quality of life questionnaires. These strategies to aid patient recall will be tested in a single centre, randomised controlled trial comparing alternative strategies of assessing change in patient reported outcomes with treatment in an acute care hospital in Australia. The parallel groups comprise COPD patients commencing pulmonary rehabilitation and COPD patients waiting to commence the program. We will compare three strategies to improve patient recall with the standard approach of patient’s completing the questionnaires at each study time point. These new strategies in addition to current practice are (1) the use of tablet technology’s audio-visual functionality and (2) the provision of patient access to their baseline questionnaire responses prior to completion of questionnaires at follow-up. Additionally, we are assessing the feasibility of using these patient strategies to improve patient recall when completing self-report PROs questionnaires in a busy acute care hospital. Feasibility will be assessed according to the National Institute for Health Research (NIHR) feasibility study criteria (Table 1) [18].

**Table 1:** Feasibility assessment for recall study

Feasibility item	Record
Willingness of clinicians to refer patients to the study	Number of referrals to study
Willingness of participants to be involved in the study	Number of patients approached but chose not to participate
Number of eligible patients	Number of patient who are eligible based on criteria
Number of participants that dropped out of study	Number of participants who withdrew and reasons or died
Follow-up rates	Number of participants who completed time 2 questionnaires
Length of time to complete recruitment of participants	Time in months
Length of time to administer intervention	Time in minutes
Length of time to analyse data	Time in days

### Participants, interventions and outcomes

#### Study setting

Participants will be recruited from the COPD patient population who attend a respiratory service at Liverpool Hospital, Sydney, a large tertiary hospital in Australia. The study has been accepted and recorded on the Australian and New Zealand Clinical Trial registry (ANZCTR No. 12618001605280) and lists the single site where this study is being conducted.

#### Eligibility criteria

To be eligible for the study, adults must have a medical diagnosis of COPD and be able to read to year 7 standard to ensure unaided completion of the quality of life questionnaires. Patients with COPD who have significant medical comorbidities that require ongoing acute care will be excluded from participating in the study. Any COPD patient with significant neurological and cognitive impairment such as medically diagnosed dementia will also be excluded. After confirming the patient’s eligibility, study information will be given to patients by the research assistant. Once the patient has had time to review the study information, consent will be sought (Additional file 1). On gaining consent, the patient will be enrolled into an arm of the study and interviewed by the research assistant and data collected. Participants will be re-interviewed 1 month later from the time of their first interview with the research assistant.

#### Interventions

**Fig. 1:**
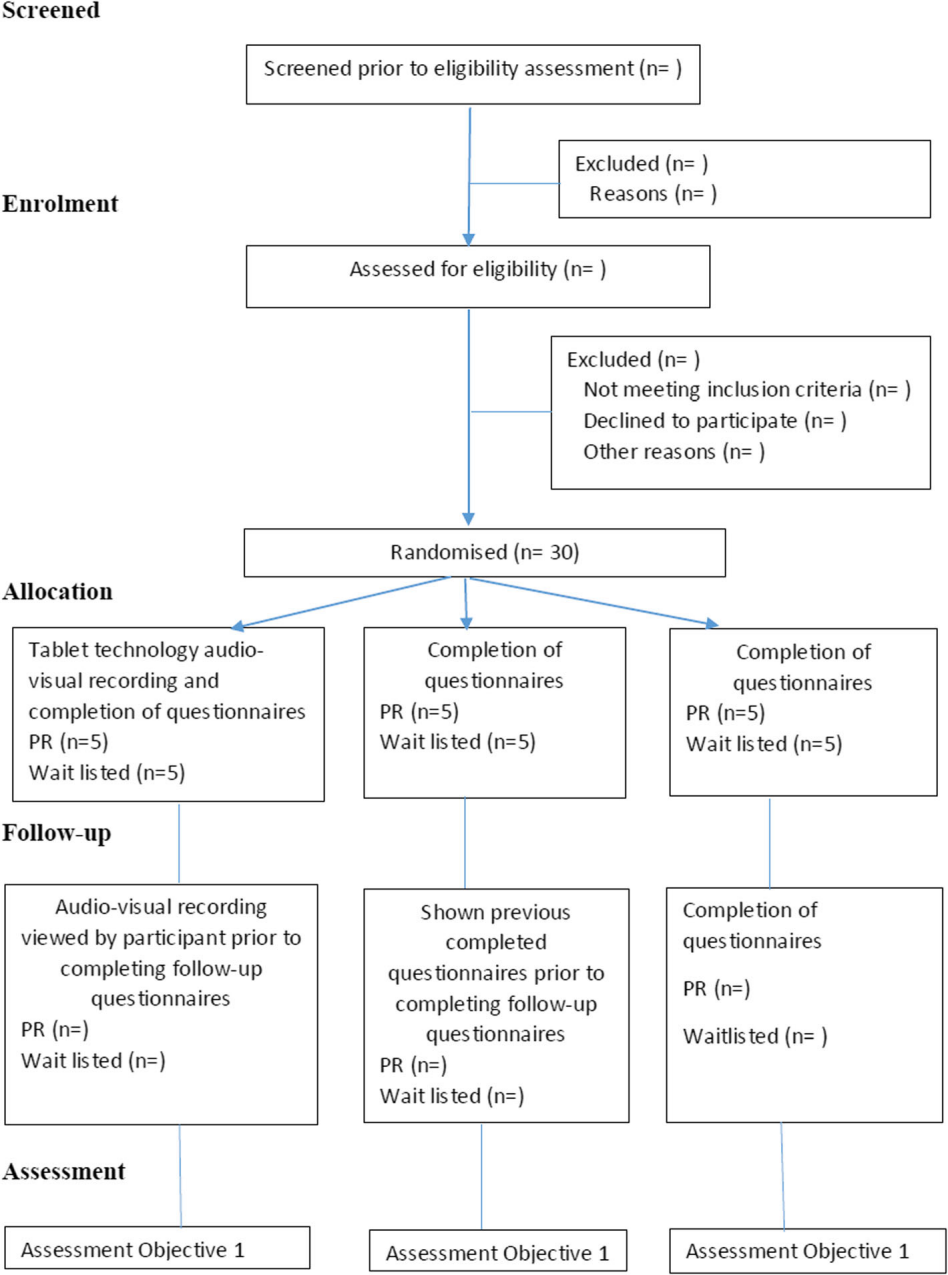
Study participant numbers in each group and study interventions protocol

Study participants will be randomly allocated to one of the following strategies to enhance patient recall (Fig.1):

1. Tablet computer technology audio-visual recording: As part of recording participants at baseline, they will be asked to respond to questions on the health questionnaires which they have just completed by self-report. Participants will be shown this video 1 month later prior to the completion of the follow-up questionnaires.
2. Shown hard copy of baseline responses: Participants will be provided with hard copies of their baseline responses to review immediately prior to completing follow-up questionnaires.
3. Standard practice: Participants will be required to complete the health questionnaires at baseline and 1 month later without access to their previous responses or an audio-visual recording and receive no feedback on their baseline responses.

Clinical care will not be affected and the clinical team will continue the medical management of the patient during the timeframe of the study. The investigator team members are not part of the clinical or pulmonary rehabilitation teams. Patients who become clinically unwell and unable to attend the follow-up appointment will be included in the analysis.

#### Outcomes

There are two outcomes associated with this study; evaluating the feasibility of conducting this study in an acute tertiary admission hospital using the National Institutes for Health Research’s feasibility criteria and testing the hypothesis related to the difference in quality of life indices. The feasibility outcome for this study is the ability to recruit participants who are wait-listed or attending the pulmonary rehabilitation program, administer the interventions in a timely manner and the ease of conducting and completing this study in a busy clinical environment without disruption to the pulmonary rehabilitation service or patient care. The secondary outcome of this randomised controlled trial is concerned with the impact of the patient recall strategies on the quality of life questionnaires’ responsiveness. Specifically, we are measuring the magnitude of statistical difference of quality of life between the three arms of the study.

#### Participant timeline

COPD patients attending the respiratory service of a large tertiary hospital who are commencing or waiting to commence pulmonary rehabilitation will be invited to consider taking part in the patient recall study. Pulmonary rehabilitation staff receive the patient referrals from the hospital’s medical and nursing staff. The pulmonary rehabilitation clinical staff will initially approach potential participants, and if the patient verbally consents, the research assistant will further assess the patient for eligibility and their willingness to participate in the study. Patients will be given the study’s approved patient information sheet to read prior to being asked if they will continue with the consenting and enrolment process with the research assistant. All engagement and communication with participants will be conducted in a separate private office away from the clinical area. Once the patient is consented and enrolled, the research assistant will assign the participant to an arm of the study using the randomisation code. Baseline data will be collected and the randomised intervention will commence. At the second and final data collection point, the intervention will be implemented and follow-up data collected. Once 30 participants are enrolled and their data collected at baseline and follow-up appointments, the study’s data collection will end.

#### Sample size

For this pilot randomised control trial and feasibility study, we did not generate a sample size. Thirty (30) adults with COPD, who are either commencing or wait-listed for pulmonary rehabilitation, will be enrolled in this study. The data generated from this study will be used to estimate important parameters that are required to design a larger randomised controlled study to evaluate the role audio-visual recordings using tablet computer technology may play in assisting patient’s recall of their previous health state.

#### Recruitment

Our pulmonary rehabilitation service is the largest in the health district, which is located on the hospital campus. COPD patients referred to pulmonary rehabilitation can wait up to 6 weeks before commencing the program. All COPD patients, with a referral to or attending the pulmonary rehabilitation program, will be informed about the study and invited to consider participating.

### Assignment of interventions

#### Allocation

For this pilot study, participants will be allocated to one of three recall strategies; tablet technology audio-visual recording, reviewing their previous responses or standard practice (Fig. 1).

#### Sequence generation

Eligible participants will be randomised 1:1:1 assigned ratio to one of the three intervention arms in both the pulmonary rehabilitation and wait list groups using the computer-generated randomisation code.

#### Allocation concealment mechanism

The research assistant will hold the allocation code and the study investigators do not have access to the code or the participant assignment information. Clinical staff and the study investigators will be unaware of a participant’s assignment to an intervention arm of the study.

#### Implementation

Our biostatistician generated a computerised randomisation allocation code. The research assistant will assign study participants to one of the three interventions in this study based on the generated allocation code.

#### Blinding

Participants and the medical team responsible for their treatment and care will be blind to the assignment of the intervention a participant receives. As this study does not interrupt, change or affect clinical care, the unblinding of participants will not be permissible.

### Data collection, management and analysis

#### Data collection methods

Consenting, enrolled participants will be assessed at baseline and again 1 month later at the follow-up appointment. The research assistant will record the patient’s demographic information, clinical history, comorbid conditions, medical therapy and past activity and exercise history. The participant will complete the following patient-reported outcome questionnaires at baseline and again 1 month later.

- European Quality of Life tool that has five dimensions and questions (EQ5D) [19]
- The Health Survey Short Form with 36 questions (SF36) [20]
- St Georges Respiratory Questionnaire (SGRQ) [21]
- Hospital Anxiety and Depression Scale (HADS) [22]

Functional measurements will also be completed at baseline and 1 month later. These include six-minute walk test and lung function testing comprising forced expired volume over 1 s.

Feasibility of conducting this study will be assessed over the course of the study and the following data recorded (Table 1).

- Willingness of clinicians to refer patients to the study
- Willingness of participants to be involved in the study
- Number of eligible patients
- Count of participant’s who drop-out
- Follow-up rates
- Length of time to complete recruitment (months)
- Length of time (min) needed to administration the questionnaires
- Length of time (weeks) required to complete data analysis

#### Data management

All data will be stored on a password-protected computer in a university research office with security swipe access. Participant data will be entered into SPSS version 23 study database from hardcopy completed questionnaires and study forms that record the functional assessments. Data will be audited after the first 15 participants’ data have been enrolled and their data entered onto the database and at the end of the study prior to data analysis to ensure data quality and accuracy. The audit will be a comparison between the hard copy of the completed questionnaires and the entered data.

#### Statistical methods

Demographic and clinical information will be assessed using descriptive statistics measuring frequencies, means and standard deviations. We will measure the within-subject change in PRO questionnaire scores over the 1-month period. The difference in mean change between those who had pulmonary rehabilitation and those who remained on the waiting list over this 1-month period will be calculated. Finally, the magnitude of this difference (responsiveness) of these scores will be compared between groups. Our hypothesis is that the magnitude of the difference (responsiveness) will be greatest in those who saw the audio-visual recording.

Feasibility will be reported using descriptive statistics such as frequencies, rate, means and standard deviations to interpret whether it is feasible to conduct a larger study in this busy clinical environment.

### Monitoring

#### Data monitoring

This study does not have external funding and, as such, there are no competing interests with a sponsor. A data monitoring committee has not been constituted for this pilot and feasibility study. Should the results of this study suggest the need for a larger randomised control trial is warranted to evaluate the role for the use of table technology audio-visual functionality in patient recall, a data monitoring committee will be formed to provide oversight of the study and the associated study procedures. As this is a small pilot feasibility study, interim analyses will not be undertaken. Data analyses will commence after the final enrolled participant in the study completes their follow-up questionnaires and an audit of database is undertaken to confirm the data entered to be accurate.

#### Harms

The hospital’s research and ethics committee has reporting mechanisms for the adverse events, expected and unexpected and serious adverse events. For this study, it is unlikely that there will be adverse events as treatment and clinical care do not form part of this study; however, the reporting mechanisms including template forms and procedures are available to the investigators of this study.

#### Auditing

The audit process for this study pertains to procedures for identifying potential participants, delivery of the intervention and data entered into the database. A random audit of study procedures and the database will be carried out after the first 15 participants are enrolled. Any breaches of the protocol or errors in the database will be addressed within 1 week of the audit and the hospital’s research and ethics committee will be notified according the hospital policy and procedure.

### Ethics and dissemination

#### Research ethics approval

Ethical approval has been granted from the Research and Ethics Committee of South Western Sydney Local Health District (HE14/214) as a low and negligible risk study and reciprocal Human Research Ethics Committee’s approval from Western Sydney University (H10841).

#### Protocol amendments

The hospital’s research and ethics committee has procedures for protocol amendments and these include template forms and processes to be completed by the investigators and submitted to the committee for approval.

#### Consent or assent

The research assistant is responsible for consenting potential participants after they have read and indicated they understood the study information to ensure informed consent. Other forms of consent, assent or authorised surrogates, will not be acceptable for enrolment into this patient recall study.

#### Confidentiality

Data will be de-identified and the research assistant will hold the master file with the participant’s name and contact details in a secure locked file cabinet in a university office that has security swipe access to enter. All de-identified data will be entered into the study’s database on a password-protected computer. Hard copies of completed questionnaires will have an assigned number with no personal identification details being recorded, and these completed questionnaires will be stored in a locked filing cabinet in a secure office at the university.

#### Declaration of interest

The study’s investigators have no conflict of interests to declare.

#### Access to data

All investigators will have access to the final dataset and there are no contractual agreements that limit access to this dataset.

#### Ancillary and post-trial

In the study’s patient information sheet, the issue of distress is raised and potential action indicated. For example, “If you suffer any distress or psychological injury as a result of this research project, you should contact the research team as soon as possible. You will be assisted with arranging appropriate treatment and support”.

#### Dissemination policy

On completion of the study, planned dissemination of the results will comprise a national conference presentation and manuscript publication.

## Additional file

Additional file 1: Informed consent materials. (DOC 110 kb)

## Data Availability

Not applicable.

## Abbreviations

6MWT: Six-minute walk test
COPD: Chronic obstructive pulmonary disease
EQ5D: European Quality of Life 5 Dimensions
FEV_1_: Forced expired volume over 1 s
GM: Guy marks
HADS: Hospital Anxiety and Depression Scale
JD: Joseph Descallar
PRO: Patient-reported outcomes
SF36: Health Survey Short form 36 questions
SGRQ: St Georges Respiratory Questionnaire
SJ: Stephen Jan
SMS: Sheree M S Smith
