# Supplementary material for: An investigation of methods to improve recall for the patient-reported outcome measurement in COPD patients: a pilot randomised control trial and feasibility study protocol
Source: Pilot Feasibility Stud. 2019 Jul 18;5:92. doi: 10.1186/s40814-019-0475-9 (PMC6637538; doi:10.1186/s40814-019-0475-9)
Supplement: Supplementary file 1 — Informed consent materials. (DOC 110 kb) [file 40814_2019_475_MOESM1_ESM.doc]

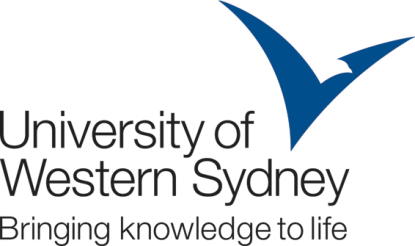


***[Liverpool Hospital]***

**CONSENT FORM**

[To be used in conjunction with a Participant Information Sheet]

**Improving Recall in Patient Reported Outcomes**

1. I,................................................................................................................. of................................................................................................................

agree to participate in the study described in the participant information statement set out above ***(attached to this form).***

1. I acknowledge that I have read the participant information statement, which explains why I have been selected, the aims of the study and the nature and the possible risks of the investigation, and the statement has been explained to me to my satisfaction.
2. Before signing this consent form, I have been given the opportunity of asking any questions relating to any possible physical and mental harm I might suffer as a result of my participation and I have received satisfactory answers.
3. I understand that withdraw from the study at any time without prejudice to my relationship to the **University of Western Sydney and <Liverpool Hospital>.**

1. I agree that research data gathered from the results of the study may be published, provided that I cannot be identified.
2. I understand that if I have any questions relating to my participation in this research, I may contact Professor Sheree Smith on telephone (02) 4620 3532 who will be happy to answer them.
3. I acknowledge receipt of a copy of this Consent Form and the Participant Information Statement.

**Signature of participant Please PRINT name Date**

**_________________________ _______________________ _______________**

**Signature of witness Please PRINT name Date**

**_________________________ _______________________ _______________**

**Signature of investigator Please PRINT name Date**

**_________________________ _______________________ _______________**
